# Dual-energy computed tomography in acute ischemic stroke: state-of-the-art

**DOI:** 10.1007/s00330-020-07543-9

**Published:** 2020-12-14

**Authors:** Stephanie Mangesius, Tanja Janjic, Ruth Steiger, Lukas Haider, Rafael Rehwald, Michael Knoflach, Gerlig Widmann, Elke Gizewski, Astrid Grams

**Affiliations:** 1 Department of Neuroradiology, Medical University of Innsbruck, Innsbruck, Austria; 2 Neuroimaging Research Core Facility, Medical University of Innsbruck, Innsbruck, Austria; 3 NMR Research Unit, Queens Square MS Centre, Department of Neuroinflammation, UCL Queen Square Institute of Neurology, Faculty of Brain Science, University College London, London, UK; 4 Department of Biomedical Imaging and Image Guided Therapy, Medical University of Vienna, Vienna, Austria; 5 Institute of Neurology, University College London, London, UK; 6 Department of Radiology, University of Cambridge, Cambridge, UK; 7 Department of Radiology, Medical University of Innsbruck, Innsbruck, Austria; 8 Department of Neurology, Medical University of Innsbruck, Innsbruck, Austria

**Keywords:** Stroke, Brain ischemia, Tomography, X-ray computed, Hemorrhage, Thrombosis

## Abstract

**Abstract:**

Dual-energy computed tomography (DECT) allows distinguishing between tissues with similar X-ray attenuation but different atomic numbers. Recent studies demonstrated that this technique has several areas of application in patients with ischemic stroke and a potential impact on patient management. After endovascular stroke therapy (EST), hyperdense areas can represent either hemorrhage or contrast staining due to blood-brain barrier disruption, which can be differentiated reliably by DECT. Further applications are improved visualization of early infarctions, compared to single-energy computed tomography, and prediction of transformation into infarction or hemorrhage in contrast-enhancing areas. In addition, DECT allows detection and evaluation of the material composition of intra-arterial clots after EST. This review summarizes the clinical state-of-the-art of DECT in patients with stroke, and features some prospects for future developments.

**Key points:**

*• Dual-energy computed tomography (DECT) allows differentiation between tissues with similar X-ray attenuation but differentatomic numbers.*

*• DECT has several areas of application in patients with ischemic stroke and a potential impact on patient management.*

*• Prospects for future developments in DECT may improve treatment decision-making.*

## Introduction

The benefit of dual-energy computed tomography (DECT) compared to single-energy computed tomography (SECT) in acute ischemic stroke has been documented in several studies [1–5]. DECT reliably differentiates between tissues with similar X-ray attenuation but different compositions, with regard to atomic numbers [6], and further extrapolates virtual monochromatic series of a certain tube voltage [7].

Initially, DECT was designed for coronary imaging, as it provides not only visualization of stenosis but also high accuracy for the detection and characterization of coronary plaques [8–10].

For neuroradiological applications [6, 7, 11–17], four major different DECT techniques are available: (1) dual-source X-ray tubes with different tube voltages, 80–100 kV and 140 kV, performing simultaneously (Siemens); (2) single-source rapid voltage switching between two tube voltages (GE); (3) a single-source split beam, where the X-ray beam is split into two different energy spectra that differ in table feel direction (Siemens); and (4) a dual-layer detector with simultaneous data acquisition of the low- and high-energy dataset (Phillips) [6, 18, 19].

To date, there are only two published review articles that describe the application of DECT in cerebral or cerebrovascular diseases from 2015 and 2016 [18, 20] and one on emergency neuroimaging from 2016 [21]. Recently published studies on the application of DECT in acute ischemic stroke have not yet been recapitulated. The aim of this literature review is to provide an overview of the clinical state-of-the-art of this rapidly evolving technology.

## Methods

A comprehensive search strategy was developed combining the following major themes: “Dual-energy computed tomography” AND “acute ischemic stroke.” PubMED and EMBASE were separately searched using the following terms: “Dual Energy Computed Tomography” or “Dual Energy CT.” To this first screening we added either “Haemorrhage” or “Ischemia” or “Infarction.” All studies available in the English language were included, regardless of the publication date, through 5 July 2020.

After excluding papers focused on other medical conditions, case reports, animal model studies, or biomolecular markers, and, after a cross-reference analysis, we identified 25 pertinent studies that satisfied our inclusion criteria (19 DECT applications in acute ischemic stroke, and additional six for limitations of DECT applicability). We added another 34 articles for background information (25), comparison to alternative approaches (4), and future outlook (5).

## Clinical background

With routinely performed SECT “brain window” (BW) series, early signs of cerebral infarctions can be visualized between 3 and 6 h after onset [22, 23]. Following endovascular (EST) or intravenous stroke therapy, technical success needs to be assessed to determine patient outcome. With EST becoming the standard therapy in large-vessel occlusions, further requirements for post-interventional imaging have emerged. A disruption of the blood-brain barrier (BBB) may lead to either hemorrhage or contrast staining following intra-arterial contrast agent (CA) administration, both of which are challenging to differentiate on SECT BW scans [18].

Furthermore, the detection of residual arterial clots or early arterial re-thrombosis after EST in follow-up scans is of the utmost interest for further treatment decisions. However, clots might not be visible with SECT BW imaging or CT angiography following CA administration.

While many of the abovementioned imaging challenges could be mastered with MR imaging, MRI scans are more time-consuming, sometimes not available, and their applicability hampered by patient-related contraindications such as claustrophobia. Consequently, “optimized” CT imaging for acute ischemic stroke in emergency settings is urgently needed for treatment decision and prognostic determination.

DECT further allows dose reduction, which, due to the non-negligible radiation exposure of patients, has recently become a critical concern.

However, CT perfusion (CTP) and CT angiography (CTA) are also described to be accurate in detecting acute ischemic stroke [24, 25] and, therefore, a comparison between one-stop CTP/CTA and DECT is needed.

We performed literature research, which focused on studies that applied DECT for the imaging (i) of acute ischemic stroke, (ii) after EST, and (iii) of acute cerebral arterial clots.

## Differentiation between hemorrhage and iodine staining

In a phantom study with increasing hematocrit (5–85%) to model hemorrhage, virtual non-contrast (VNC) attenuation of spectral detector CT differentiated accurately between blood and iodinated contrast mixtures in contrast to conventional image attenuation [26].

**Fig. 1:**
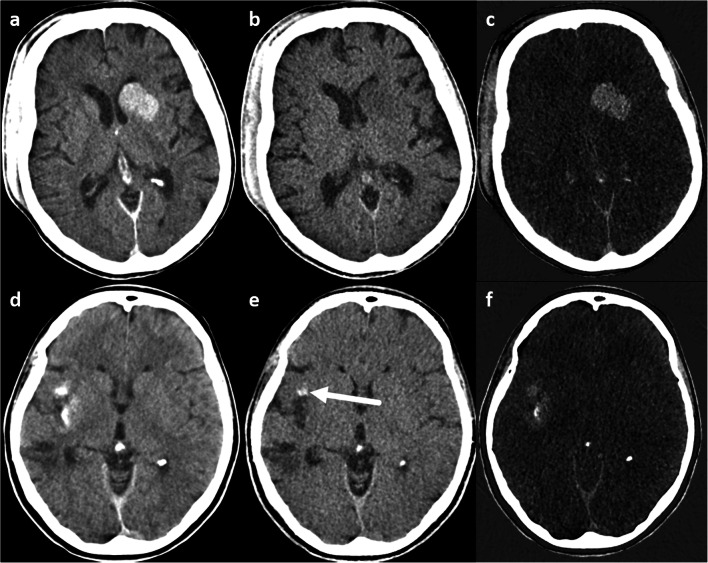
Brain window (**a**, **d**), virtual non-contrast (**b**, **e**), and iodine map (**c**, **f**) series. The upper row shows a hyperdense area in the left basal ganglia on the brain window and iodine map, but not in the virtual non-contrast series, representing contrast staining; the lower row shows a hyperdense area in the right insular cortex of the brain window and the iodine map series, which is only partly hyperdense in the virtual non-contrast series (arrow), representing both contrast staining and hemorrhage

Most clinical studies on the application of DECT in ischemic stroke investigated the differentiation between hemorrhage and iodine contrast staining due to BBB disruption, especially after EST. While iodine map series (IM) depict contrast-enhancing areas, VNC represents “unenhanced” imaging (Fig. 1).

The evaluation of VNC and IM series of 14 patients allowed the differentiation between hemorrhage and contrast enhancement with an accuracy of at least 91% in 28 hyperdense areas, as confirmed on follow-up scans [27]. In 40 patients, 148 hyperdense areas were investigated and correctly classified with 100% sensitivity, 84.4–100% specificity, and 87.2–100% diagnostic accuracy [28]. In 19 patients, VNC and IM series achieved an 89% accuracy in comparison to BW series, with 63% [29]. Hyperdense areas of 48 patients after EST were assigned to contrast-enhancement or hemorrhage with no false-positives or false-negatives [30]. A reliable distinction was achieved throughout using 80- and 140-kV tube voltage DECT. However, patient numbers are fairly low given the increasing performance of EST worldwide. Therefore, studies on larger cohorts with subjective and objective rating scales are needed to ultimately confirm the high accuracy of DECT.

Hemorrhage and contrast enhancement might, however, not be the only cause for hyperdense areas.

The diagnostic quality regarding intraparenchymal hemorrhage (IPH) and hypodense parenchymal lesions (HPL) of 58 patients with conventional image (CI) and virtual monoenergetic image (VMI) reconstructions, ranging from 40 to 120 keV from dual-layer detector CT (DLCT), were objectively assessed by ROI-based measurements, contrast-to-noise ratio (CNR) calculation, and a 5-point Likert scale. The improved depiction of HPL and IPH on VMI was greatly dependent on the type of pathology and its location. Hypodense lesions in white matter (WM) and hyperdense lesions in the gray matter (GM) were better visualized at higher/120 keV, while hyperdense lesions in the WM and hypodense lesions in the GM were better visualized at low/40 keV [31].

A recent meta-analysis of DECT demonstrated excellent diagnostic performance in terms of differentiating acute ICH from contrast staining and small calcifications. However, it was suggested that publication bias may lead to overestimation of diagnostic performance, warranting large-scale, prospective cohort studies [32].

## Imaging of acute stroke

The second most emphasized improvement of DECT is visualization of acute cerebral infarctions or hemorrhagic infarctions with prior contrast application.

“Non-contrast-weighted sum images” resembling BW and VNC imaging in a series of 58 patients after EST of the middle cerebral artery (MCA), 24 h prior to DECT (80/140 kV) scans, achieved an improved detection of infarction with VNC imaging compared to BW imaging, based on the ASPECTS score, despite moderate inter-rater concordance [1, 2]. For 28 patients with acute cerebral infarction, “true non-contrast” (resembling a BW series), VNC, “brain edema” (BE), and a 24-h follow-up series were reconstructed with 90-/150-kV DECT within 4 h after onset and classified by the ASPECTS score, with good inter-rater reliability. The BE series was reconstructed by changing the relative dual-energy contrast value according to the ratio of density differences between GM and WM on the 90-/150-kV series. The BE imaging could detect brain edema and predict future infarction volumes more accurately than the BW or the VNC series [3]. A so-called X-map was reconstructed from 80-/150-kV DECT scans of six patients within 20 h after stroke onset by changing the parameters of the DE “bone marrow” application. The X-map focused on the mean densities of the GM and the WM, and was then subjectively compared to a “simulated standard CT” and follow-up diffusion-weighted magnetic resonance imaging (DWI-MRI) series. All ischemic lesions were detected with X-maps, and none was detected on the BW series [4]. In a continuative study on 80-/150-kV DECT in 11 patients, iterative beam-hardening correction and settings in the “bone-marrow” application were modified to achieve improved suppression of GM/WM contrast. This advanced “X-map 2.0” allowed improved correlation with infarction extent on DWI [33].

**Fig. 2:**
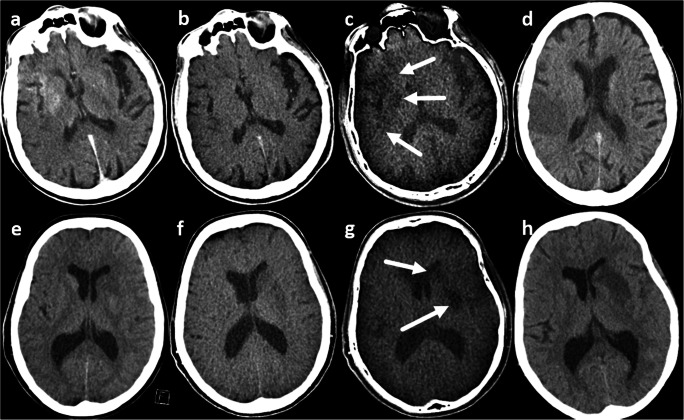
Brain window (**a**, **e**), virtual non-contrast (**b**, **f**), edema map (**c**, **g**), and follow-up (**d**, **h**) brain window series. The upper row shows no infarction demarcation in the brain window or the virtual non-contrast series, but a clear demarcation on the edema map (arrows), corresponding to the follow-up series; the lower row shows only a slight infarction demarcation in the left basal ganglia on the brain window and the virtual non-contrast series, but a clear demarcation on the edema map (arrows), corresponding to the follow-up series

In a study of 100-/140-kV DECT scans immediately after EST in 46 patients, the DE software settings were changed to focus on mean densities of infarction/brain rather than hemorrhage/iodine contrast by inserting mean densities of infarctions instead of hemorrhage on the 100- and 140-kV series. In addition, relative dual-energy contrast was adapted according to the mean density decrease of non-infarcted brain tissue from the 100–140-kV VNC series. Tissue contrast was determined quantitatively by contralateral-to-infarction ROI density measurements, with high inter-rater reliability. Infarction volumes were measured on three DECT series and follow-up imaging. The highest cortex-to-infarction contrast and the best prediction of future infarct size were achieved with EM compared to BW and VNC (Fig. 2). The infarction location did not affect the detection rate [34].

Better image quality was achieved on 80- and 100-keV extrapolated, monoenergetic series from DECT scans of 30 patients with early posterior fossa infarctions compared to SECT and there was a trend toward a better infarction detectability on the 100-kV series [5].

Compared to SECT, different DECT techniques can, therefore, improve the detection of early infarction.

However, while X-maps yield promising results, some problems may result in false-positive or false-negative findings. DECT images bear smearing artifacts near the brain–skull boundary, and X-map values present large inter-patient and intra-patient variations, especially with regard to the GM. Some authors argue that X-maps cannot replace the simulated standard CT for the diagnosis of acute ischemic stroke [4, 35].

In addition, patients with symptom onset up to 24 h before CT were examined [2, 4], at a time point in which infarctions could be depicted with BW. Furthermore, objective measurements were not applied in some studies. Instead, subjective rating scales were assessed [2–5, 33]. Some studies feature small patient numbers [4, 33]. However, none of the studies in EST patients provided thrombolysis in cerebral infarction (TICI) scores prior to or after therapy, for reasons which remain unknown. Therefore, it is unclear whether the absence of this score should be interpreted as a marker of technical therapy success, or as a study limitation.

In a study on simulated conventional CT images after EST, the Hounsfield unit (HU) values with IPH on VNC were significantly higher than in its absence (cutoff 80 HU), and HU values in the presence of delayed IPH were substantially higher than without it (cutoff 78 HU) [36].

VMI monochromatic 99-keV images yielded the best balance between infarction and normal brain parenchyma for the evaluation of acute MCA brain infarction and were not inferior to 70-keV images [37].

## Prediction of infarction development or future hemorrhagic transformation in contrast-enhancing areas

**Fig. 3:**
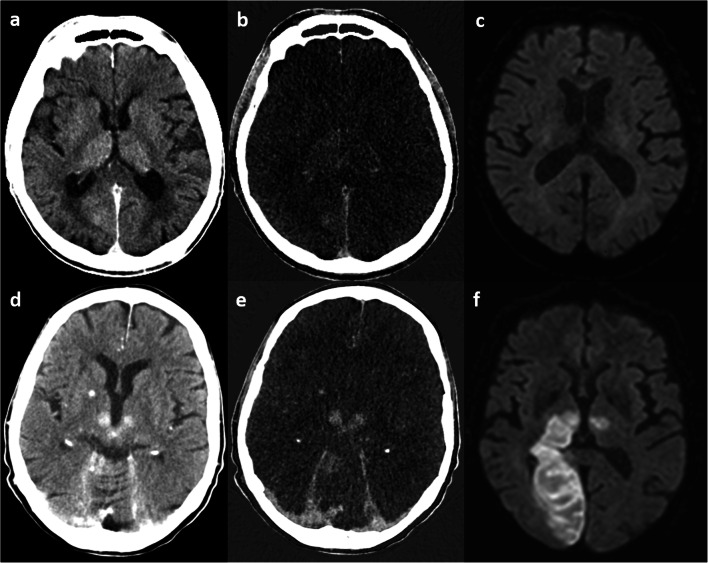
Brain window (**a**, **d**), iodine map (**b**, **e**), and diffusion-weighted follow-up MRI (**c**, **f**) series. The upper row shows a slight iodine staining in both thalami and the right occipital lobe on the brain window and the iodine map series, with no future infarction; the lower row shows more pronounced iodine staining in both thalami and the right upper cerebellum, with later infarction development

Contrast-enhancing areas on DECT (100/140 kV) immediately after EST in 132 patients were found in 32 and hemorrhage in 53 patients. Follow-up imaging was either CT or MR imaging. Subjective assessment by an uncertain amount of raters revealed both contrast enhancement (odds ratio (OR) 11.3) and hemorrhage (OR 10.4) to be associated with a poor clinical outcome (modified Rankin scale 3–6), even if complete recanalization was achieved. In addition, patients with contrast-enhancing areas displayed a higher rate of delayed hemorrhagic transformations (OR 4.5) [38]. To investigate under which circumstances contrast-enhancing areas would transform to infarctions or delayed hemorrhage, the DECT scans (100/140 kV) of 20 patients with 44 contrast-enhancing areas who underwent EST were analyzed and ROI density measurements were performed on BW, VNC, and IM imaging by two raters, withhigh inter-rater reliability. Areas that transformed to infarctions revealed higher densities on IM, representing a higher amount of contrast enhancement (Fig. 3), with a cutoff value of 34.1 HU, compared to areas that did not transform (area under the curve (AUC) 0.99). For the prediction of delayed hemorrhage, the AUC was 0.78 [39]. On the DECT (80/140 kV) images of 85 patients immediately after EST, maximum iodine concentration was measured by two raters, if iodine staining was present (*n* = 54). In areas with an iodine concentration > 1.35 mg/dl, later development of hemorrhage was more likely, with an AUC of 0.89 [40].

These studies suggest that the presence or amount of contrast staining after EST may have predictive value for the clinical outcome. However, in some studies, clinical parameters were not considered and follow-up imaging was inconsistent with regard to time interval and imaging method [38–40].

## Clot imaging

DECT may be beneficial for the visualization of intracranial arteries with clot material after prior contrast administration, compared to SECT. In the first in vivo study, it was concluded that an additional BW may be omitted. As with the VNC reconstructions from CT angiography, hyperdense arteries can be detected; however, no evaluation of the VNC with regard to the detection of early infarctions was performed, which is at least equally important in the acute stages [41]. In the second in vivo study, there was no comparison of arteries with residual clot material to the “gold-standard,” TICI scoring (thrombolysis in cerebral infarction), although conventional angiography studies were available [42]. The results about clot composition of artificial clots have yet to be verified in vivo [43, 44].

DECT can be applied to differentiate intra-arterial CA from clots. To date, in vivo studies about the detection of intracranial arterial clots are sparse.

Intra-arterial clot detection on a VNC series reconstructed from DECT (80/140 kV) angiography was equal to that on a BW/true non-contrast series in 30 patients with stroke and hyperdense-artery signs and 30 controls. That series achieved a 93% accuracy upon application of a subjective rating scale and objective ROI density measurements within arteries with clot material, contralateral arteries without clot material, and the arteries of controls [41].

**Fig. 4:**
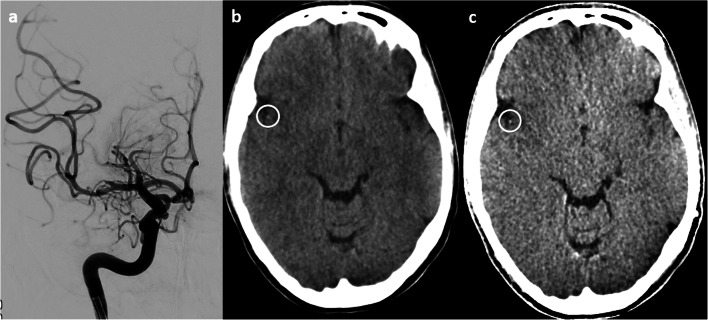
Conventional angiography (**a**), brain window (**b**), and virtual non-contrast (**c**). A residual clot of the M2 part of the right middle cerebral artery known from the final angiography series after thrombectomy, seen as hyperdense dots on the brain window and the virtual non-contrast series (circles)

In 10/16 patients with EST of large intracranial arteries and immediate post-interventional DECTs (100/140 kV), residual peripheral clots were found on the digital angiography series on the BW, VNC, and IM protocols. Arteries with clot material were identified in 9/10 cases on the VNC only, while the absence of clots was correctly rated in 5/6. Arteries with clot material were significantly denser than perfused arteries on the VNC only (Fig. 4) [42].

## DECT in the context of CTA and CTP

To date, comparative studies between DECT and CTP or CTA technology regarding the abovementioned advantages of DECT compared to conventional CT and MRI in the application of acute ischemic stroke remain sparse.

CTA provides the treating physician of patients who present with acute ischemic stroke with advantages that help in clinical decision-making, such as detailing the presence of occlusion, the extent of thrombus, and neurovascular anatomy [45], with known drawbacks, including radiation, risk of contrast-induced nephropathy, and the time required to perform a CTA [45].

One advancement in this field is one-stop CTP/CTA technology, which has been widely used in clinical diagnosis and prognostic evaluation of patients with cerebral ischemic stroke. Especially in an emergency situation, apart from demarcating the ischemic phase and range, one-stop CTP/CTA technology can also determine the location and range of embolus and, therefore, assist with clinical diagnosis and treatment.

Collateral status, as assessed with different scoring systems, is an important factor that determines outcome in acute ischemic stroke. Upon the application of single- and multi-phase CTA (spCTA and mpCTA) and compared to CTP parameters, collateral status evaluated on spCTA was shown to suffice for outcome prediction and decision-making in patients with ischemic stroke, potentially obviating further imaging modalities like mpCTA or CTP [25].

Despite little evidence, CTP might be more accurate than NCCT with an accuracy similar to that of CTA in detecting acute ischemic stroke, thus demonstrating a potential treatment benefit of CTP in select stroke patients [24].

Novel 4D CTA scan protocol has been introduced, including a non-enhanced CT, dynamic CTA with reconstructed arterial and venous phases, perfusion of the whole brain, and CTA of the carotid arteries in a single acquisition using a 320-row area detector CT scanner. This approach involves a unique contrast injection technique and an acceptable radiation exposure dose in patients who present with acute ischemic stroke, and also uses model-based iterative reconstruction (MBIR), time summing reconstructions (time-maximum-intensity projection, tMIP), and subtraction techniques to optimize the results [46].

CTA is also used to determine a potential thrombus residue after thrombectomy, which is significant for the clinical outcome [47], as re-occlusion is more common in cases with residual thrombus [48].

DECT is valuable in the detection of clot persistence or early re-thrombosis without the necessity for additional contrast administration, but its relevance for the prediction of outcomes remains to be determined in further studies [42].

Further studies are needed to compare the value of DECT and CTA, as well as CTP.

## Future aspects

A newly developed technique, multi-energy CT (MECT), employs novel photon-counting detectors in an image-based material decomposition task.

Photon-counting detector models with multiple energy bins achieve a performance comparable to DECT at 100 kV/Sn 140 kV. With the use of photon-counting detectors in dual-source protocols, however, the performance can be improved above the level of a single, realistic photon-counting detector and also dual-source DECT [15].

Greater GM/WM differentiation, compared to conventional CT, with excellent intra-reader and inter-rater reproducibility, was achieved, which was linked to higher soft tissue contrast and 12.8–20.6% less image noise [15].

A spectral photon-counting detector (PCD) CT for the evaluation of the major arteries of the head and neck achieved significantly higher image quality scores with lower image noise and fewer image artifacts compared to conventional single-energy CT scans using energy-integrating detectors (EID).

Intra-arterial artifacts on EID images were not depicted on PCD images due to beam hardening. Iodine maps achieved a 20.7% higher CNR compared to non-spectral PCD, and VMI at 70 keV provided CNR similar to that of non-spectral images. Consequently, photon-counting CT might potentially improve the image quality of carotid and intracranial CT angiography compared to single-energy EID CT [49].

A recently proposed new method is to extract the effective atomic number (Zeff) and electron density (ρe) from DECT images based on a Karhunen-Loeve expansion (KLE) of the atomic cross-section per electron. An effective atomic number map provides, therefore, a quantitative approach to material differentiation by analyzing attenuation changes as a function of energy [6, 50]. This approach has already found clinical applications, such as the advanced detection of lung perfusion defects caused by pulmonary embolism on an effective atomic number map compared to an iodine map [6]. Another neuroradiological application is the prediction of brain tumor histology, where intracranial neoplasms were found to be separated into three major groups: gliomas, meningiomas, and metastases [51]. The useful value of z-maps appears to be the percentage change in the effective atomic number following contrast enhancement [52]. Potentially, the calculated effective atomic number of tissue in every voxel might also be useful for clot differentiation. Further studies are required to investigate the potential of this approach in the workup of acute ischemic stroke.

Despite the benefits of DECT for material differentiation, material decomposition suffers from magnified noise from two CT images of two separate scans, potentially leading to degradation of image quality. This might be due to the fact that implemented algorithms exhibit suboptimal decomposition performance, which fail to fully depict the mapping relationship between DECT images and basic materials, especially under noisy conditions. Improvements are expected from techniques such as iterative reconstruction to enhance image quality and artificial intelligence, which will likely boost material decomposition. Convolutional neural networks have recently become an important technique in medical imaging, including modeling of data coupling to perform image domain material decomposition for DECT. The proposed approach was shown to lead to higher decomposition quality in noise suppression on clinical datasets compared to those using conventional schemes [53].

## Limitations

Given the different technical approaches of DECT imaging as provided by different companies, imaging data may differ marginally and hamper their comparability in clinical applications. Hence, the results of the studies reported in the present review may not be repeatable with different scanner types and different scanner generations [53, 54]. Large multi-center studies would be needed that would include different scanners to confirm whether the achieved results are applicable when using alternative techniques. However, some findings have been validated multiple times in different studies even with different scanning techniques, thus emphasizing the robustness of the DECT method.

While DECT material decomposition is usually successful in neuroradiology, a major limitation is that the distinction is limited to up to three preselected materials.

Using a three-material decomposition algorithm to obtain VNC and iodine (or calcium) overlay images, DECT was successfully used in 90.2% of 72 cases for the differentiation of CA staining and/or extravasation from IPH, calcium from IPH, and for metal-artifact reduction and angiographic assessment. However, a fourth material, for example, parenchymal calcifications, may confound the analysis. Material decomposition is further impaired by artifacts, such as beam hardening, metallic streak, or saturation effects [55].

The presented studies, however, provide an important hint for possible future applications of DECT, and about how the latest CT technologies may change our daily diagnoses to achieve more accurate results.

While this review is not intended to assess the various scanner technologies and imaging methods, Table 1 provides an overview of which scanner technology achieved specific research advancements by describing technical terms with kilovolt pair settings.

**Table 1:** Overview of the different scanner technologies (described by kV pair settings) and their described differential diagnostic imaging value

Imaging	kV pair setting (tube voltage)	References
Differentiation of hemorrhage from iodine staining	80/140 kV	Gupta et al (2010) [27],Phan et al (2012) [28], Tijssen et al (2014) [29], Morhard et al (2014) [30]
Acute stroke	80/140 kV	Gariani et al (2015) [36]
	90/150 kV	Mohammed et al (2018) [3]
	80/150 kV	Noguchi et al (2017) [4], Taguchi et al (2018) [33]
	100/140 kV	Grams et al (2018) [34]
	80/100 kV	Hixson et al (2016) [5]
Prediction of infarction development or future hemorrhagic transformation in contrast-enhancing areas	100/140 kV	Renu et al (2015) [39], Djurdjevic et al (2017) [40]
	80/140 kV	Bonatti et al (2018) [41]
Clot imaging	80/140 kV	Winklhofer et al (2017) [44], Brinjikji et al (2017) [42]
	100/140 kV	Grams et al (2015) [45], Brinjikji et al (2017) [42]
	Spectral detector CT (120 kV)	Borggrefe et al (2017) [43]

Third-generation dual-energy scanners outperform earlier generations and ongoing improvements may be expected [53, 54, 56–58]. Standardization of methods will allow comparable imaging between different centers.

## Conclusion

The currently most intriguing clinical application of DECT in stroke patients is the differentiation between hemorrhage and contrast staining after prior iodine contrast application and arterial clots can be reliably visualized with a VNC series after iodine contrast administration.

Future research should focus on potential differences related to technically varying DECT imaging approaches between vendors. Candidates for clinical implementation in the near future are MECT, PCD, effective atomic number and electron density Zeff, and artificial intelligence.

## Abbreviations

AUC: Area under the curve
BBB: Blood-brain barrier
BE: Brain edema
BW: Brain window
CA: Contrast agent
CNR: Contrast-to-noise ratio
DECT: Dual-energy computed tomography
DLCT: Dual-layer detector CT
DWI-MRI: Diffusion-weighted magnetic resonance imaging
EID: Energy-integrating detectors
EST: Endovascular stroke therapy
FR: Fibrin-rich
GM: Gray matter
HU: Hounsfield unit
IM: Iodine map series
IPH: Intraparenchymal hemorrhage
MCA: Middle cerebral artery
MECT: Multi-energy CT
OR: Odds ratio
PCD: Photon-counting detector
RBC: Red blood cell
SECT: Single-energy computed tomography
TICI: Thrombolysis in cerebral infarction
VMI: Virtual monoenergetic image
VNC: Virtual non-contrast
WM: White matter
